# Migration of small ligands in globins: Xe diffusion in truncated hemoglobin N

**DOI:** 10.1371/journal.pcbi.1005450

**Published:** 2017-03-30

**Authors:** Polydefkis Diamantis, Oliver T. Unke, Markus Meuwly

**Affiliations:** Department of Chemistry, University of Basel, Basel, Switzerland; Baltimore, UNITED STATES

## Abstract

In heme proteins, the efficient transport of ligands such as NO or O_2_ to the binding site is achieved via ligand migration networks. A quantitative assessment of ligand diffusion in these networks is thus essential for a better understanding of the function of these proteins. For this, Xe migration in truncated hemoglobin N (trHbN) of *Mycobacterium Tuberculosis* was studied using molecular dynamics simulations. Transitions between pockets of the migration network and intra-pocket relaxation occur on similar time scales (10 ps and 20 ps), consistent with low free energy barriers (1-2 kcal/mol). Depending on the pocket from where Xe enters a particular transition, the conformation of the side chains lining the transition region differs which highlights the coupling between ligand and protein degrees of freedom. Furthermore, comparison of transition probabilities shows that Xe migration in trHbN is a non-Markovian process. Memory effects arise due to protein rearrangements and coupled dynamics as Xe moves through it.

## Author summary

Binding and transport of ligands in proteins is essential, in particular in globular proteins which often exhibit internal cavities. In truncated Hemoglobin N (trHbN) these cavities are arranged as a network with particular connectivities. The present work supports the notion that ligand diffusion in trHbN is an active process and coupled to the protein dynamics. Furthermore, transition probabilities between neighboring pockets depend on the location from where the ligand entered the transition, which is typical for non-Markovian processes. Hence, ligand migration in trHbN exhibits memory effects due to dynamical coupling between the protein and ligand motion.

## Introduction

The interplay between protein and ligand degrees of freedom is of great interest in understanding protein function including catalysis, signaling or transport. Both, ligand binding and transport between internal localization sites often leads to small changes in the functionally relevant protein structure and fluctuations around it. Changes in structure and fluctuations constitute a way to store information in proteins, for example through the concept of configurational entropy. [1, 2] The transfer of such information between spatially separated regions is essential for transducing signals in chemical and biological systems. One concrete manifestation of this is allosteric communication in proteins whereby information is transferred over distances of the size of a protein. [3–7] Allostery involves a cascade of information exchange processes which can be initiated by uptake of an exogenous ligand. This raises the question whether and if yes, in which way and on what time scale ligands in proteins influence “information flow” in proteins. Starting from a “system-bath” perspective it appears natural that ligands in proteins are active entities beyond their mere chemical role in chemical transformations.

Evolution has developed a range of mechanisms to transmit information across a protein following specific triggering events such as binding of a ligand or absorption of a photon. A recent review of the current state of understanding of allostery pointed out that the “new view” of allostery, which is based on population shift, is actually reminiscent of the 1965 model put forward by Monod, Wyman and Changeux (MWC). [3] This view of allostery underlines the importance of equilibrium fluctuations for the function of allosteric proteins. In a protein-ligand context these equilibrium fluctuations may be subject to localization of a ligand in different pockets and following different pathways. Allosteric control involves several key steps which eventually lead to biological function. Often, the first step is a local structural change, induced by ligand binding. Next, this information is propagated through the protein network. The time scale for this ranges from picoseconds to microseconds, highlighting the large variation in temporal and spatial scales involved in allostery. In a later phase, structural changes lead to affinity changes which complete the chain of events. Hence it is of genuine interest to better understand the mechanistic basis of local structural changes induced by ligand localization and migration, and the time scales on which they occur and relate them to the fate of the system on longer time scales. [8–11]

The binding and reaction of small gas phase ligands like oxygen (O_2_) or nitrogen monoxide (NO) with heme proteins is involved in processes that are essential for the physiological function of living organisms. The ligand binding sites of heme proteins are buried. Therefore, ligand migration from the outside towards the active site involves ligand diffusion, often occurring through a network of connected, internal sites. Extensive research has shown that such networks of internal cavities (pockets, packing defects) exist particularly in globular proteins, and has been confirmed for heme containing proteins including myoglobin (Mb) [12–14], cytochrome b*α*3 oxidase [15, 16], dimeric hemoglobin [8, 14], or truncated hemoglobins N and O [17–19].

The physico-chemical (thermodynamic and kinetic) properties of ligand migration and reaction with heme proteins have been extensively investigated. Besides facilitating ligand diffusion towards the binding site of the protein, ligand migration networks have also been suggested to detain ligand molecules in their pockets, either for storage [13, 16], or for subsequent detoxification [18]. Nonetheless, further investigation on the structural and temporal nature of ligand motion in the protein is required to better understand whether and, if so, how protein and ligand motion are coupled.

In Mb, it has long been suggested that the binding of small ligands to specific pockets affects the internal motions and conformational substates adopted by the protein [12]. This should also apply to other heme proteins exhibiting a similar ligand migration network. In fact, recent computational work on i) NO and O_2_ migration in truncated hemoglobin N of Mycobacterium Tuberculosis [20–22], ii) CO migration on myoglobin [23] or iii) Benzamidine in the trypsin inhibitor [24] point in a similar direction: the conformational dynamics of the protein and the ligand (probe) are coupled and, depending on the localization of the ligand within the protein (or at its surface), the conformational dynamics of the protein changes.

**Fig 1:**
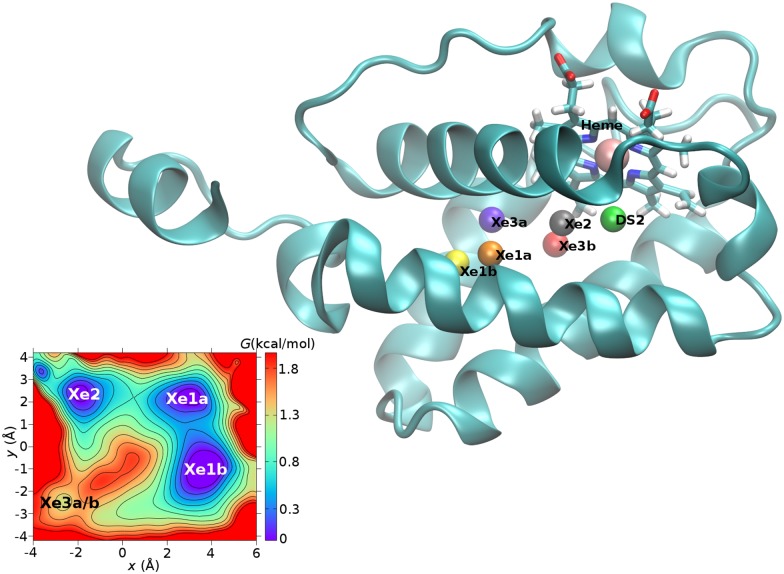
TrHbN and important pockets. Main Figure: The crystal structure of trHbN, with the heme group in licorice, Xe1a/b, Xe2, Xe3a/b and DS2 pockets as spheres. Inset: 2-dimensional cut through the free energy surface along the plane defined by Xe2, the saddle points between Xe1a and Xe1b, and between Xe3a and Xe3b, respectively.

In this work, the nature of ligand migration in truncated hemoglobin N (trHbN, see Fig 1) of Mycobacterium Tuberculosis has been investigated using molecular dynamics (MD) simulations. The migration pathways for NO and O_2_ in trHbN have been extensively characterized experimentally [17, 18] and computationally [20–22, 25–29]. TrHbN is an ideal protein for such an investigation as earlier studies [20, 21, 25–27, 29] have revealed that the free energy barriers between different pockets are low (1 to 2 kcal/mol) which allows to extensively sample transitions between them. Xenon (Xe) has been selected as the ligand for this study as it has been used in the experimental determination of binding pockets within trHbN [18] (see Fig 1) and cytochrome b*α*3 oxidase [16]. In addition, Xe has been suggested to be a good mimic of O_2_ [16], a physiologically relevant ligand of trHbN and other heme proteins. Furthermore, evidence from theoretical studies on myoglobin [13] suggests that Xe migration networks in heme proteins share the same pockets and pathways with natural ligands, such as O_2_, NO and CO.

In the present work ligand migration and the interplay between ligand and protein dynamics is investigated from a ligand perspective and at atomic resolution. In particular, the question whether, how and on what time scale the motion of a diffusing Xenon atom influences protein dynamics is quantitatively assessed. Furthermore, we analyze potential memory effects in the transition dynamics between neighboring pockets depending on where the Xenon atom entered a transition path of interest.

## Methods

### Molecular dynamics simulations

All simulations were performed using the CHARMM suite of programs [30] with the CHARMM22 force field [31] and the van der Waals radius of Xe was *R*_min_ = 2.25 Å which is close to a radius of 2.24 Å used in previous work [13]. For water, the TIP3P model was used [32]. All simulations were carried out with a timestep of 1 fs and a cutoff of 14 Å was used for the non-bonded interactions, and SHAKE was used for constraining the bonds involving hydrogen atoms [33, 34]. Long-range electrostatic interactions were treated with Ewald summation [35, 36], employing 96, 64 and 64 grid points along the *x*−, *y*− and *z*−axis, respectively.

The B subunit of a crystallized structure of the trHbN dimer (Protein Data Bank (PDB) Index: 1S56 [18]) was used as the starting structure with one Xe atom initially placed in either the Xe1, Xe2 or Xe3 pocket, which are the focus of the present work. The monomer was solvated in a pre-equilibrated periodic water box (71Å, 56Å, 56Å). The system was then heated and equilibrated at 300 K. Two different equilibration procedures were considered. In the first, Xe was weakly restrained with a force constant of 5 kcal/mol/Å^2^ to its initial pocket (Xe1, Xe2 or Xe3) which helps to better sample the region of interest (Xe1, Xe2, Xe3). Simulations based on a second equilibration scheme (with no restraints imposed on Xe) confirmed that restraints do not lead to artifacts. In both cases, frames were extracted every 2.5 ps and used as starting points for the production phase (*NVE*) simulations. In total 725.5 ns of simulations were run and analyzed.

### Free energy surface for Xe migration

An effective 3-dimensional free energy surface for Xe migration in trHbN was constructed using the Xe coordinates from ≈ 200 ns of simulation. A regular grid with spacing of 1 Å along the *x*−, *y*− and *z*−direction was employed for this. The Helmholtz free energy *G*(*x*, *y*, *z*) at each grid point was computed according to *G*(*x*, *y*, *z*) = −*RTln*(*P*(*x*, *y*, *z*)) where *P*(*x*, *y*, *z*) is the probability of observing Xe in point (*x*, *y*, *z*), *R* is the universal gas constant and *T* is the temperature in K. A smooth free energy surface was then constructed by interpolating the discrete points of *G*(*x*, *y*, *z*) using 3-dimensional cubic B-splines [37].

### Clustering

#### *k*−means clustering

In *k*−means clustering [38] a collection of data points is decomposed into disjoint sets with respect to the distance of each point from a number of geometrical centers, each defining a cluster. Every point is assigned to the cluster center closest to it. In the system investigated here, the centers of mass of 9 main pockets (Xe1a, Xe2, Xe3, Xe4, Xe1b, DS2, ENT, IS1, PDS) were provided as an initial input. For each pocket, the coordinates of its center of mass were determined from the amino acids forming it [29]. The algorithm was used using two cut-off distances *r*_min_ = 1.75 Å and *r*_min_ = 6.20 Å, similar to a previous analysis of ligand migration in trHbN with *k*−means [21]. The inner cut-off defines a sphere within which Xe positions should be used for re-calculating the centers of the clusters during the analysis, whereas the outer cut-off decides which frames should not be assigned to any of the 9 clusters. These points (outliers) were assigned to a tenth cluster, named “Else”.

#### Robust Growing Neural Gas algorithm

Xe coordinates were also clustered using the Robust Growing Neural Gas (RGNG) algorithm [39]. Contrary to *k*−means, RGNG does not require an initial guess for the centers of the clusters. The algorithm is very robust due to (i) its insensitivity to the presence of outliers, and (ii) its ability to auto-determine the optimal number of cluster centers for a given dataset based on a minimum description length criterion [40].

#### Final clustering

Based on the free energy surface, the position of each pocket center was refined using conjugate gradient descent starting from the pocket center coordinates obtained by the clustering techniques (*k*−means, RGNG). In addition, the minimum energy paths (MEPs) for relevant transitions within the (Xe1, Xe2, Xe3) states including their substates were also determined. For each transition, the corresponding MEP was obtained using conjugate peak refinement [41]. The pockets (defined by pocket centers) and the transition regions (defined by saddle points) determined on the free energy surface of trHbN were used for the final clustering of the Xe coordinates.

## Results/discussion

Before discussing the transition dynamics between neighboring pockets and their dependence on the trajectory history, the relevant states for the present work are discussed.

### Characterization of the states

Table 1 provides a summary of the percentage occupation of each state (i.e. the fraction of total simulation time spent by Xe in a particular pocket), according to *k*−means clustering, RGNG, and the final clustering (clustering with respect to both, pocket centers and transition regions).

**Table 1:** Clustering of Xe position.

Pocket	*k*−means (%)	RGNG (%)	Final Clustering (%)
Xe1a	13.1	12.9	11.5
Xe1b	31.5	33.0	33.4
Xe2	6.9	6.9	6.4
Xe3a	5.9	6.1	5.5
Xe3b	7.0	7.2	7.5
Xe4	1.5	—	0.5
DS2	15.2	15.1	15.2
ENT	2.9	—	0.9
IS1	1.5	—	1.1
PDS	0.6	—	0.0
Else	13.9	18.8	13.6
Transition States	—	—	4.9

The fraction of total simulation time that Xe spent in each pocket (occupation) according to *k*−means clustering, the RGNG algorithm, and the final clustering. In RGNG, “Else” corresponds to *k*−means “Else” + Xe4, ENT, IS1 and PDS, which are not found by RGNG. In the final clustering, “Else” is used to cluster all points not belonging to any of Xe1a/b, Xe2, Xe3a/b, Xe4, DS2, ENT, IS1, PDS pockets or transition states. The occupation of PDS for the final clustering is 0.003%

According to Table 1, based on *k*−means clustering, the most occupied pocket is Xe1b, followed by DS2, Xe1a, Xe3b, Xe2, and Xe3a. In addition, Table 1 shows that over the course of the MD simulations, the region comprising pockets Xe1a/b, Xe2, and Xe3a/b are highly populated with an aggregated population of 64.4% of the total simulation time. The region containing Xe3 is particularly interesting as previous work showed that it is a hub of the ligand migration network of trHbN [22, 27–29]. The high occupation of the (Xe1, Xe2, Xe3) region was expected, given that the majority of MD simulations were started with Xe in any of these three pockets to increase sampling probability of the relevant transitions involving these pockets. Hence, the occupations in Table 1 are probably not representative of an equilibrium population which, however, is of no concern for the purpose of the present work. Ligand diffusion in trHbN has previously been investigated for O_2_ and was analyzed with different clustering methods, including *k*−means, kinetics-based Markov Clustering (MCL) and the locally scaled diffusion map (LSDMap) [21]. The O_2_–occupation of trHbN differs significantly from the one found in the present work for Xe a) because Xe and O_2_ interact differently with the protein, and b) because the simulations started with Xe in either Xe1, Xe2 or Xe3. Hence, for O_2_ exploration of the network started from an equilibrium distribution which was not the case here.

As an independent validation of *k*−means, pocket analysis was carried out with the RGNG algorithm. The RGNG clustering yielded six clusters which correspond to the most frequently visited Xe pockets according to *k*−means clustering, see Table 1. Compared to *k*−means, the centers of pockets Xe1a, Xe1b, Xe2 and DS2 shift by less than 1 Å. The finding of 2 clusters that both belong to the Xe3 pocket agrees with previous indications of this pocket being large and diffuse [29]. In hindsight the separation of Xe3 into Xe3a and Xe3b could already have been anticipated in an earlier investigation of O_2_ diffusion in trHbN [21]. In the present work, RGNG allows to explicitly identify two separate sub-states within the Xe3 pocket. This is similar to the situation in Mb where the Xe4 pocket is also large but contains two substates which are separated by a small but nonzero free energy barrier [42]. To distinguish these two pockets, they are referred to as Xe3a and Xe3b, respectively. These two states are separated by a 1.1 kcal/mol and 0.9 kcal/mol forward and reverse barrier, respectively. Finally, for Xe3a and Xe3b, the respective occupations are 6.1% and 7.2% in RGNG. For the (Xe1, Xe2, Xe3) region the total occupation fraction is 66.1%, which supports the 64.4% fraction obtained from *k*−means.

**Fig 2:**
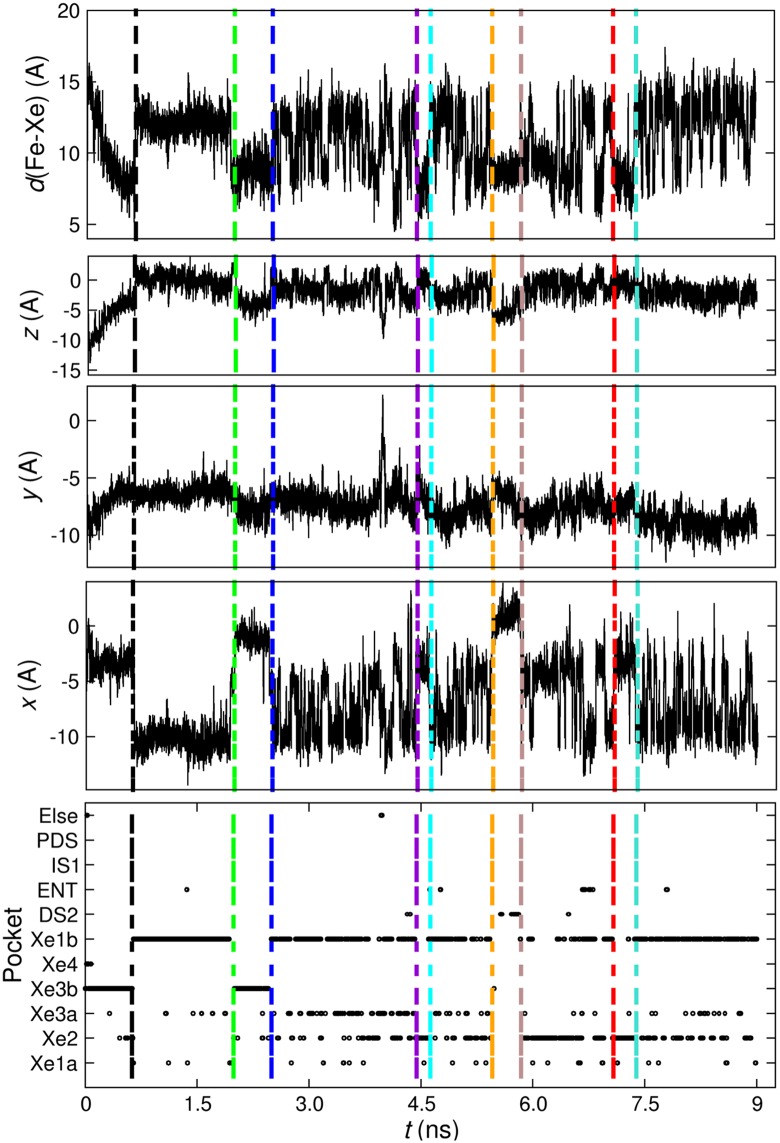
Illustration of transitions in a 9 ns trajectory. Identification of Xe transitions by tracking the evolution of the Fe-Xe distance and *x*−, *y*−, and *z*−distances from origin (Fe atom), over 9 ns of explicit MD. The selected transitions are shown via vertical dashed lines. For clarity, points belonging to transition regions (index 12) are not displayed in the bottom panel of the figure.

**Fig 3:**
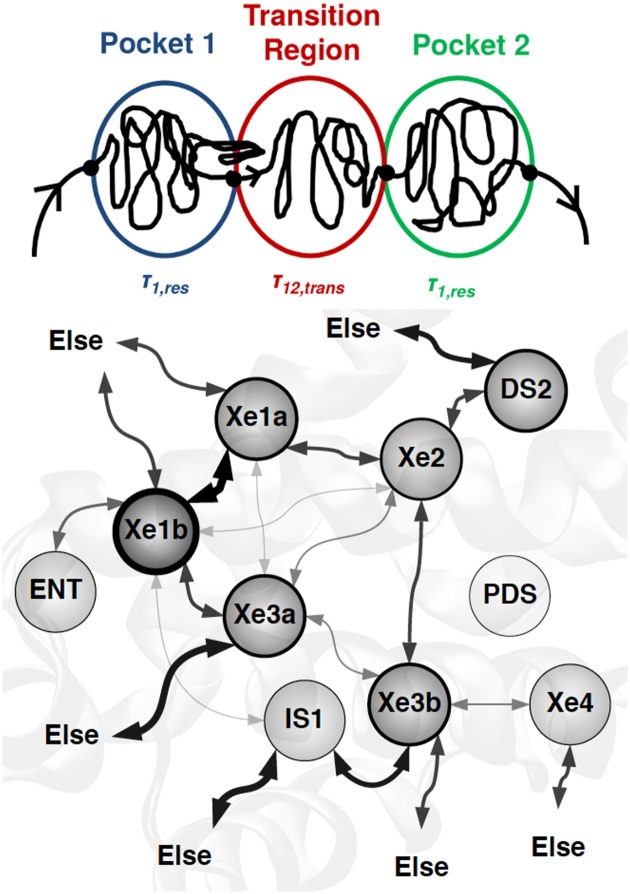
The network of Xe migration in trHbN. The top part of the figure illustrates the definition of residence times *τ*_1,res_ and *τ*_2,res_ in pockets 1 (blue) and 2 (green) and the definition of transition time *τ*_12,trans_ between the two pockets. For the network, the thicker the arrow is, the more transitions are observed between the two pockets it connects. The width of the circular borders represents the occupation probability of the respective pocket.

In order to validate the assignment of Xe positions to particular pockets, explicit time series along the Xenon *x*−, *y*−, and *z*−coordinates are compared with the discrete time series from the clustering. It is found (see Fig 2) that pocket assignments can be difficult at boundaries between regions because of the non-spherical shape of the cavities. As a consequence, an event may be assigned to the wrong state if Xe is in a transition region between neighboring states. This was indeed confirmed when visualizing *k*−means- and RGNG-clustered points in 3 dimensions. Further analysis of the clustered points would thus lead to non-realistic estimates of life times, transition times, and transition probabilities. As a consequence, additional states corresponding to transition regions were introduced to alleviate this problem (see top panel of Fig 3 for more detail).

The pockets (defined by pocket centers) and transition regions (defined by saddle points) determined on the free energy surface of trHbN were used for the final clustering of Xe coordinates for the subsequent analysis. The occupation of each pocket following this procedure is presented in the fourth column of Table 1. When transition states are included in the clustering, the total occupation of the (Xe1, Xe2, Xe3) region is 64.3% of the total simulation time, consistent with *k*−means and RGNG clustering. In addition, Xe is found in transition regions during 4.9% of the total simulation time.

### Dwell and transition time distributions

After clustering with respect to both pockets and transition regions, 19252 transitions were identified from more than 700 ns of simulation. The transitions between neighboring pockets are summarized in Table 2 and graphically represented in Fig 3. Once “states” are defined, transitions between them can be analyzed quantitatively by means of residence (“dwell”) times *τ*, their distributions *p*(*τ*) and transition times. The residence time is defined by the time Xe remains at a given basin, prior to its transition to another basin via the corresponding transition state, and the transition time is defined as the time spent by Xe in a transition region as it moves between different pocket basins, see Fig 3.

**Table 2:** Table of transitions.

	Xe1a	Xe2	Xe3a	Xe3b	Xe4	Xe1b	DS2	ENT	IS1	PDS	Else
**Xe1a**	0	391	61	4	1	1467	0	0	0	0	418
**Xe2**	396	0	110	383	0	101	348	0	8	1	3
**Xe3a**	57	103	0	129	1	326	1	1	11	1	1208
**Xe3b**	5	428	128	0	171	13	6	1	636	1	454
**Xe4**	0	0	0	175	0	0	1	0	0	0	505
**Xe1b**	1505	94	316	11	3	0	0	230	35	0	561
**DS2**	0	356	1	9	1	1	0	0	0	0	998
**ENT**	0	0	2	2	0	224	0	0	0	0	28
**IS1**	0	10	11	610	0	28	0	0	0	0	1216
**PDS**	0	0	1	0	0	0	1	0	0	0	2
**Else**	417	3	1201	462	505	543	994	28	1188	1	0

The transition matrix between initial (row) and final (column) pocket as obtained from the explicit MD simulations. A total of 19252 transitions was found.

Table 2 and Fig 3 highlight the role of the entire Xe3 pocket as a hub in the Xe-migration network in trHbN. Xe3a and Xe3b are connected to all other pockets, except for ENT. It is noted that the transition matrix is nearly symmetric. Furthermore, previous work for O_2_ migration in trHbN found barriers ranging from 0.5 kcal/mol to 1.5 kcal/mol between neighboring sites when treating O_2_ with a fluctuating charge model. [29] Given that the present simulations were initiated with Xe being in one of the Xe1a/b, Xe2 or Xe3a/b pockets, the populations of pockets outside this region do in general not correspond to an equilibrium distribution. This is, however, of no concern because the focus here is on ligand diffusion within the (Xe1a/b, Xe2, Xe3a/b) region. The stability of O_2_ in these 5 pockets is within 0.5 kcal/mol. [22] Given the total simulation time of 725.5 ns, the low barriers between the pockets and the large number of transitions observed (almost 20000), the Xe-distributions for the relevant Xe1a/b, Xe2 and Xe3a/b states are expected to be unbiased by the initial conditions.

**Fig 4:**
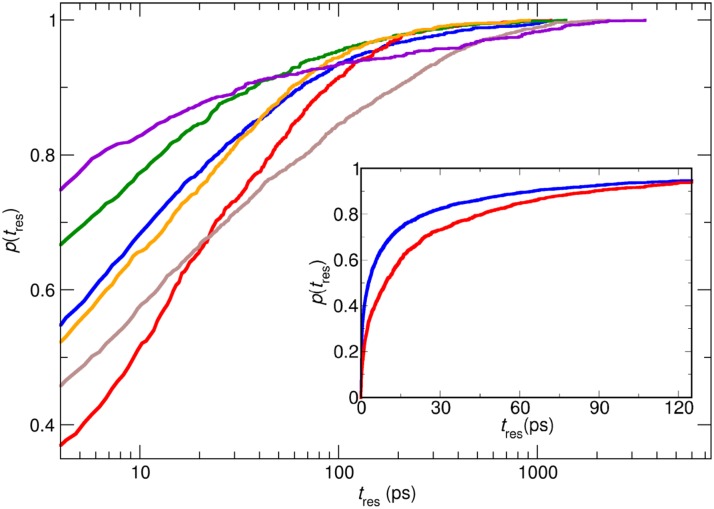
Residence time distributions. Cumulative distributions, *p*(*t*_res_) of Xe residence times, *t*_res_, in states Xe1a (blue), Xe2 (red), Xe3a (green), Xe3b (orange), Xe1b (brown) and DS2 (magenta) (double-logarithmic representation). While in all cases, up to at least 70% of the residence times are shorter than 25 ps, the logarithmic scale allows for a better illustration of the difference in the statistics of infrequently sampled long residence times for each pocket. The inset reports dwell times using a linear time axis for pockets Xe1a (blue) and Xe2 (red); 5% of the dwell times are longer than 150 ps.

The residence time distributions were analyzed for the core network (Xe1a, Xe1b, Xe2, Xe3a, Xe3b, and DS2), and is shown in Fig 4. For all states, 70% or more of the residence times are shorter than 25 ps, see inset of Fig 4. On the other hand, a small fraction of dwell times is on the 1 ns time scale or longer.

The time scale within which transitions occur was also determined. For later analysis, transition times between two particular pockets A and B were separately determined depending on the pocket C the Xe atom originated from. In other words, for a transition A→B the transition time was separately determined for C1→A→B to C*n*→A→B, where C1 to C*n* runs over all *n* pockets connected to pocket A. A→B transitions within the (Xe1a/b, Xe2, Xe3a/b) region were all found to occur on the 10 ps time scale, independently of where Xe came from before. This analysis for five different transitions is reported in Table 3. For each event, (C→)A→B, the probability was obtained by normalizing with respect to the total number of transitions from A to any other pocket, with Xe having arrived to A from C. For instance, taking Xe2 as state A and Xe1a as C*n*, (Xe1a→Xe2→B), the probabilities are 0.070 for Xe1a→Xe2→Xe1b and 0.720 for Xe1a→Xe2→Xe1a. The remaining probabilities for Xe1a→Xe2→(Xe3a,Xe3b,DS2,IS1) are 0.060, 0.078, 0.067 and 0.005 respectively, which are not shown in Table 3.

**Table 3:** Overview of selected Xe transitions.

C	A→B	Probability	Time (ps)	Counts
Xe1a	Xe2→Xe1b	0.070	6.6	27
Xe3a		0.098	6.8	10
Xe1b		0.564	4.5	53
DS2		0.014	4.8	5
Xe1a	Xe3a→Xe1b	0.138	5.9	8
Xe3a		0.211	2.4	23
Xe1b		0.142	4.8	18
DS2		0.789	4.2	247
Xe1a	Xe3a→Xe2	0.155	4.1	9
Xe3a		0.532	5.0	58
Xe1b		0.063	8.3	8
DS2		0.064	3.6	20
Xe2	Xe1a→Xe2	0.698	6.9	270
Xe3a		0.268	4.2	15
Xe1b		0.071	8.6	104
Xe1a	Xe2→Xe1a	0.720	5.3	278
Xe3a		0.186	8.9	19
Xe3b		0.116	3.2	49
Xe1b		0.191	7.2	18
DS2		0.086	5.9	30

Transition probabilities, average transition times, and number of events observed (counts) for selected transitions, obtained from the explicit MD simulations.

Table 3 shows that transition probabilities for A→B→A transitions are considerably more likely to occur than C→B→A transitions. This suggests that the physical process of ligand migration in trHbN involves appreciable dynamical coupling between ligand and protein degrees of freedom. This finding underlines the importance of trHbN fluctuations in Xe migration.

### Analysis of the Xe1a↔Xe2 transition

**Fig 5:**
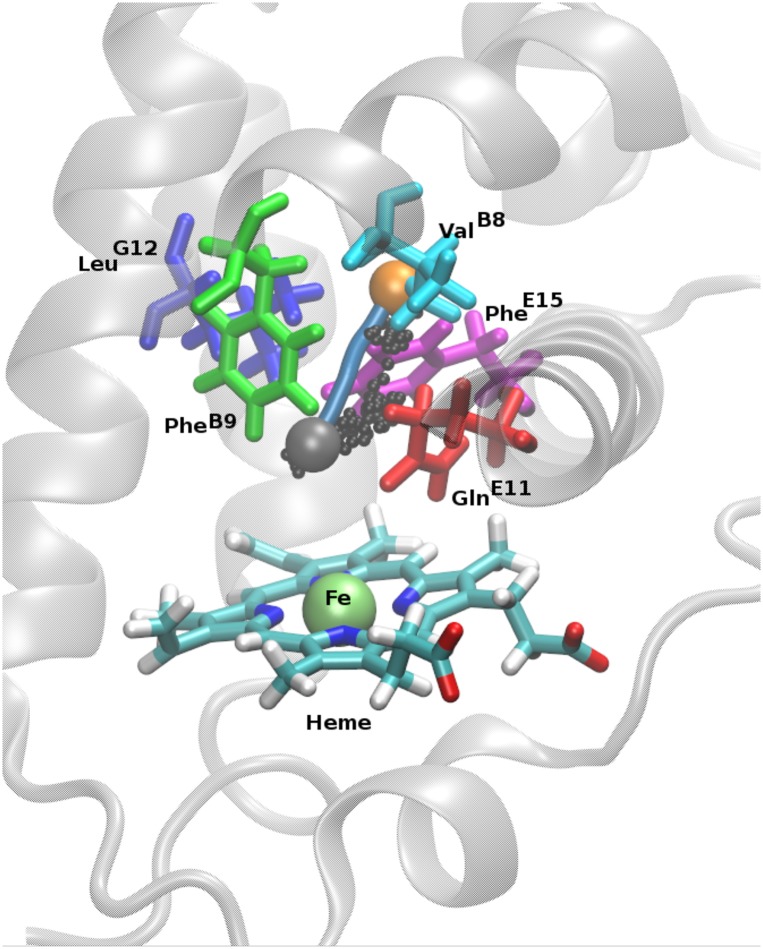
Xe1a↔Xe2 transition region. Xe1a and Xe2 pockets (orange and gray sphere, respectively), together with surrounding residues Val^B6^ (cyan), Phe^B9^ (green), Phe^E15^ (magenta), Gln^E11^ (red), and Leu^G12^(blue). The minimum energy transition path (in blue cylinder) and one particular transition path (small black spheres) are shown between the two pockets. In grey (background) the ribbon structure of the protein and in licorice the heme-unit.

In a next step the dynamical coupling between ligand and protein motion is further analyzed. For this, the Xe1a↔Xe2 transition is considered in more detail. Fig 5 shows pockets Xe1a and Xe2 along with the five amino acids that according to the MEP are involved in the Xe1a↔Xe2 transition. This transition was selected because a) it is extensively sampled, b) both Xe1a and Xe2 are connected with several other pockets, in particular, Xe1a is connected to Xe2, Xe3a and Xe1b, while Xe2 is linked to Xe1a, Xe3a, Xe3b, Xe1b and DS2, and c) Xe1a↔Xe2 is a representative transition of the (Xe1,Xe2,Xe3) core of trHbN ligand migration network.

The transition and dwell times for the Xe1a↔Xe2 transition depending on from where Xe arrived in either Xe1a or Xe2 are summarized in Tables 2 and 3. The analysis indicates that A→B→A transitions (e.g. Xe2→Xe1a→Xe2) are considerably more probable than C→B→A (e.g. Xe1b→Xe1a→Xe2) (0.698 vs. 0.071). Furthermore, the probability for a particular transition also depends on where Xe originally came from. However, transition times for Xe1a↔Xe2 do not differ significantly regardless of where the ligand came from. In a broader perspective *all* transition times are on the several picosecond time scale (3 to 8 ps) as can be seen in Table 2. Similarly, typical dwell times are 5 to 10 ps for all states, see Fig 4, although a small but potentially interesting fraction resides for up to 3.5 ns in individual states.

Pockets Xe1a and Xe2 are connected through a channel which involves amino acids Phe^B9^, Phe^E15^, Val^B6^, Gln^E11^ and Leu^G12^ (see Fig 5). The conformational space sampled by all residues involved in the channel is analyzed in the following. Only amino acid- and Xe-coordinates during the time Xe spends in the transition region (see Fig 5) are considered and analyzed. For this analysis, the protein backbone atoms, excluding the flexible N-terminus region of trHbN (first 13 amino acids) were reoriented with respect to the crystal structure of trHbN.

**Fig 6:**
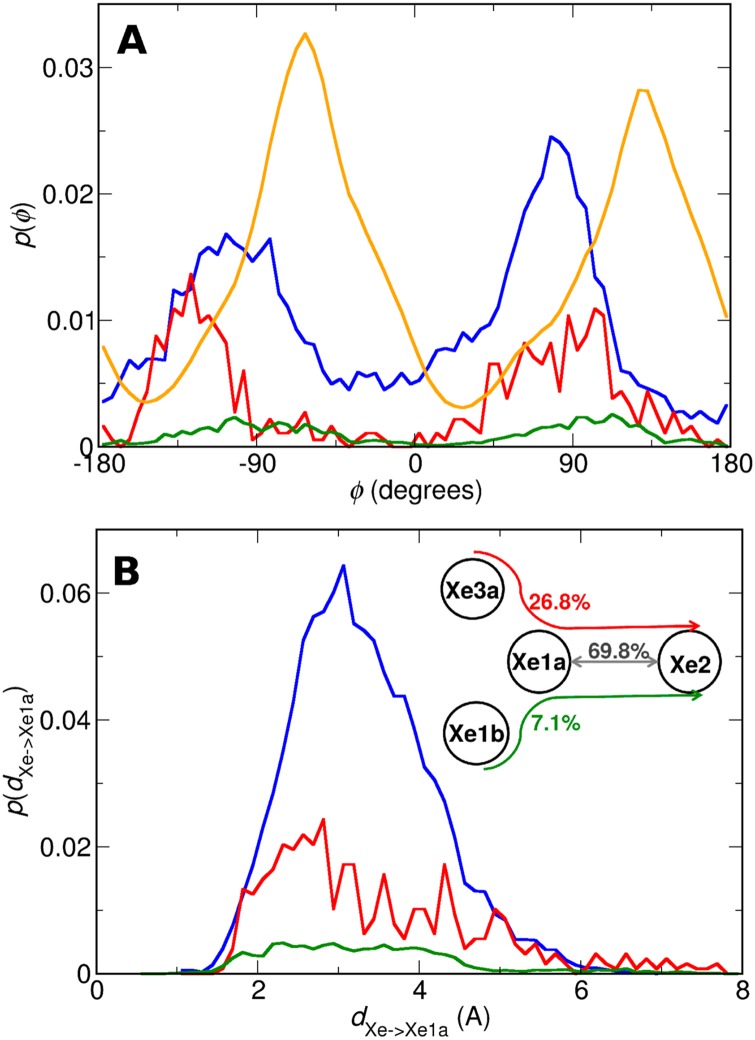
Analysis for C→Xe1a→Xe2 transitions. Distribution of the (A) C*α*-C*β*-C*γ*-C*δ*2 dihedral angle of Phe^61^ depending on state C in the transition C→Xe1a→Xe2. The equilibrium distribution (orange) is shown together with *p*(*ϕ*) for C = Xe2 (blue), C = Xe3a (red), C = Xe1b (green). (B) Xe distance from center of the Xe1a state for the Xe1a→Xe2 transitions, from explicit MD simulations. Depending on where Xe entered the Xe1a→Xe2 transition the distributions differ.

The C*α*-C*β*-C*γ*-C*δ*2 dihedral angle distribution *p*(*ϕ*) of Phe^E15^ is considered first. All distributions are normalized with respect to their individual flux as indicated in Fig 6B and Table 3 and a bin size of 5° was employed. The results show that depending on where Xe came from, *p*(*ϕ*) is different, see Fig 6A, and all distributions differ from the equilibrium distribution (orange).

The equilibrium distribution is characterized by two peaks, at around −50° and 150° respectively. On the contrary, in distributions corresponding to C→Xe1a→Xe2 transitions, these peaks are shifted. In particular, the relative populations of the two favorable orientations differ whether Xe accesses the Xe1a→Xe2 coming from Xe2 (blue) or Xe3a (red). Also, the maxima of *p*(*ϕ*) for these two cases differ by up to −100°. Moreover, besides the two peaks at −100° and 100°, the distribution for Xe2→Xe1a→Xe2 events is characterized by a significant presence of points in the intermediate region, contrary to the distributions of Xe3a/Xe1b→Xe1a→Xe2 transitions. This outcome implies a greater rotational flexibility for the Phe^E15^ ring when Xe originates from Xe2, which would indeed facilitate Xe2→Xe1a→Xe2 transitions.

In addition, we also considered the distribution of Xe distances from the initial pocket (Xe1a pocket in Xe1a→Xe2 transitions, and Xe2 pocket in Xe2→Xe1a transitions). The results for Xe1a→Xe2 transitions are illustrated in Fig 6B, while those for Xe2→Xe1a transitions are depicted in Figure S1B in S1 Text. Depending on the pocket Xe was before the transition, the magnitude and shape of *p*(*d*_Xe→Xe1a_) differs. For Xe3a/Xe1b→Xe1a→Xe2 *p*(*d*) extends to larger distances between Xe and the center from the initial pocket compared to the other two transitions.

**Fig 7:**
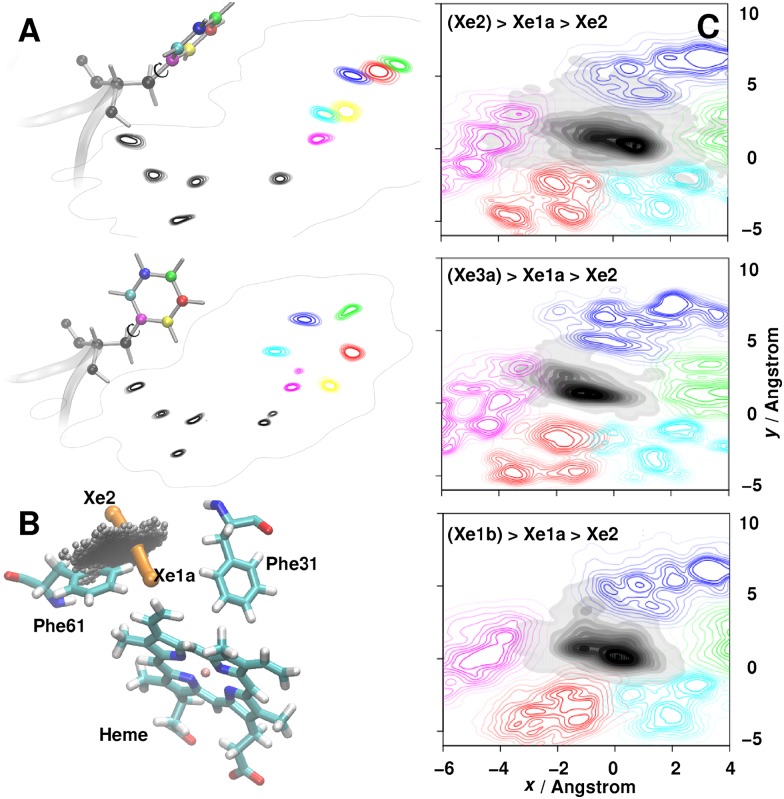
Difference in Phe^E15^ ring orientation and projection of Xe and amino acid atom positions upon C→Xe1a→Xe2 transitions. Panel A: The average orientations of the benzene ring of Phe^61^ for the two transitions (top) Xe2→Xe1a→Xe2 and (bottom) Xe3a→Xe1a→Xe2 together with the projection of each atom onto the transition plane (colored isocontours). Panel B: The projection of Xenon-positions onto the transition plane (grey)—and hence the transition plane itself-together with the Xe2→Xe1a transition (orange). Panel C: Projection of the position of Xe (greyscale) and ValB6 (cyan), Phe^B9^ (green), Gln^E11^ (red), Phe^E15^ (magenta) and Leu^G12^ (blue) atoms onto the plane containing the transition points for the Xe1a→Xe2 transition. Only transition points are included. The integrated density corresponds to 100%. Moving from inner to outer contours, each of them contains an additional 10% of the total number of points. The overall orientations of all side chains are the same throughout the Figure.

Next, the interplay between the Xe motion and the surrounding amino acids (Phe^B9^, Phe^E15^, Val^B6^, Gln^E11^ and Leu^G12^) in the transition region was analyzed. For that, a plane orthogonal to the tangent along the minimum energy path for the Xe1a↔Xe2 transition containing the saddle point as the origin was defined, see Fig 7B. All positions of the Xe and amino acid atoms were projected onto this plane and their densities are shown as isocontours in Fig 7. For clarity, filled contours and non-filled contours are used for Xe and the amino acids, respectively. The analysis finds that depending on state C before the C→Xe1a→Xe2 transition the orientation of the amino acids is different, i.e. the side chain and ligand dynamics are coupled and influence each other.

Fig 7A and 7C establish that for Xe2→Xe1a→Xe2 transitions the side chain of Phe^E15^ is oriented predominantly perpendicular to the transition plane. Contrary to that, for Xe3a2→Xe1a→Xe2 transitions it is oriented parallel to that plane. This can be clearly seen in Fig 7A where individual atom projections of the side chain ring atoms (in color) are shown. The probability distribution functions in Fig 7C underline the importance of Phe^61^ in this transition but other residues also show differing orientations depending on the transition considered. It is also noteworthy that the overlap of the Xe distributions differs for the three transitions considered. However, the present illustration may be exaggerated as all snapshots were reoriented with respect to the same protein structure (X-ray structure) which brings out the differences rather than the common features of individual transition paths for the same transition.

**Fig 8:**
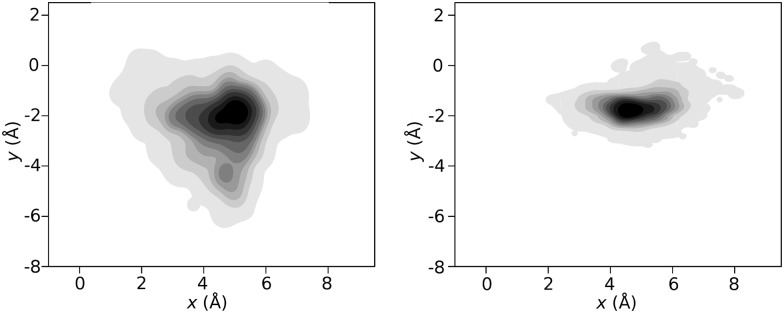
Xe position probability distributions, short vs long residence times. Comparison of probability distributions *P*(*x*, *y*) from many short dwell times (left, 5 ps) with *P*(*x*, *y*) of a simulation with a 1.2 ns dwell time (right) in the Xe1a state. The two distributions differ in shape and size because for short dwell times, Xe samples the available volume with little protein adaptation taking place whereas for long dwell times the protein is able to pack more closely around the Xe atom which typically leads to reduction of the volume of the sampled space.

Depending on the time spent by the Xe probe in a particular pocket its shape can change in different ways. Fig 8 shows that the probability distribution of Xe positions in Xe1a from many short (≈ 5 ps) dwell times differs considerably from the space sampled during one long (continuous 1.2 ns) occupation trajectory of the same pocket. This suggests that for longer dwell times the pocket has time to adapt its shape whereas for short (picosecond) dwell times the pocket does not have sufficient time to adapt.

**Fig 9:**
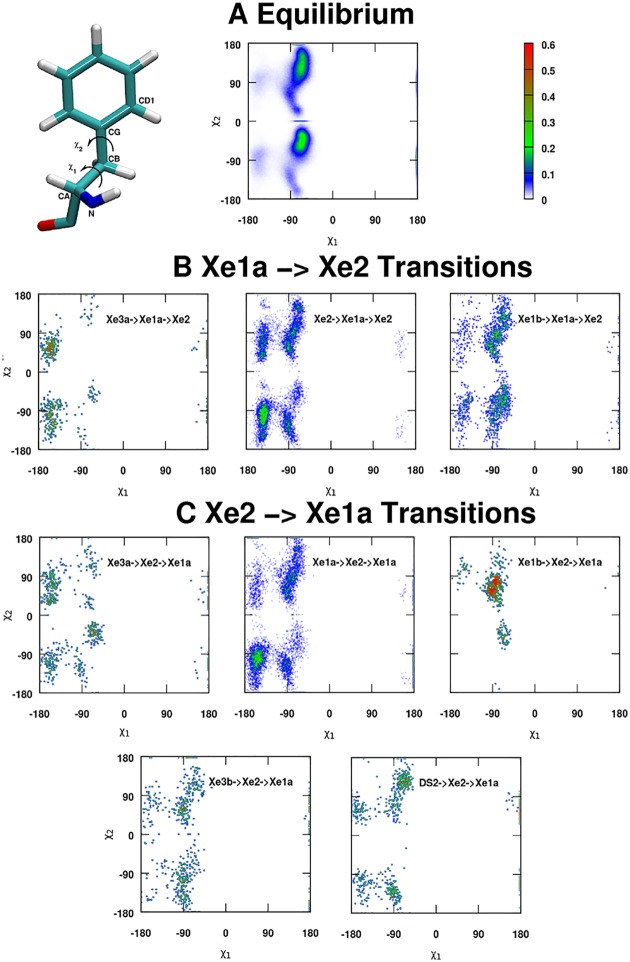
Dihedral probability distributions for Phe^E15^. 2-dimensional probability distributions *p*(*χ*_1_, *χ*_2_) of *χ*_1_ and *χ*_2_ dihedral angles upon Xe1a→Xe2 transitions, compared to the equilibrium distribution. A single Phe is reported in the left upper corner, along with the definition of angles *χ*_1_ and *χ*_2_. *p*(*χ*_1_, *χ*_2_) in panels B and C are different from the equilibrium distribution. Distributions also differ depending on where Xe came from before the transition. Characteristic examples are Xe2→Xe1a→Xe2 vs Xe1b→Xe1a→Xe2 events from panel B and Xe1a→Xe2→Xe1a vs Xe1b→Xe2→Xe1a events from panel C.

Finally, the orientation of the Phe^E15^ side chain as defined by the (*χ*_1_, *χ*_2_) angles is analyzed for Xea1↔Xe2 transitions depending on state C. The equilibrium distribution (see Fig 9A) exhibits pronounced maxima at *χ*_1_ = −90° and *χ*_2_ = (145, −45)°. Due to the small number of events for certain transitions (see Table 3) the probability distribution functions *p*(*χ*_1_, *χ*_2_) are not converged. Nevertheless, they show rather characteristic structures. For the Xe2→Xe1a→Xe2 transition (270 transitions, well sampled) *p*(*χ*_1_, *χ*_2_) exhibits a clear shift of the maximum occupation to (*χ*_1_ = −145°, *χ*_2_ = 145°). Hence, no new state is found compared to the equilibrium distribution, but the occupation of the states changes. Conversely, for Xe1b→Xe1a→Xe2 transition (104 transitions, well sampled) the distribution is more reminiscent of the equilibrium distribution. Finally, for Xe3a→Xe1a→Xe2 (15 transitions, undersampled) the majority of the distribution is along *χ*_1_ = −145° which differs from the equilibrium distribution.

For the reverse transition Xe2→Xe1a similar observations can be made. The most sampled transition is Xe1a→Xe2→Xe1a (278 transitions) for which *p*(*χ*_1_, *χ*_2_) confirms the finding for the Xe2→Xe1a→Xe2 transition. All other transitions are sampled less than 50 times and hence not particularly well converged. It is found that for the Xe3a→Xe2→Xe1a, the distribution resembles that for Xe3a→Xe1a→Xe2, whereas the remaining transitions are more similar to the equilibrium distribution. Hence, overall it is found that for the best sampled transitions the distribution functions *p*(*χ*_1_, *χ*_2_) differ in a characteristic fashion from the equilibrium distribution, whereas for other transitions the distributions can be more similar to *p*_eq_.

These observations establish that depending on from where Xe enters the Xe1a↔Xe2 transition region the channel-forming amino acids are arranged differently. Ultimately, this strongly supports the notion that protein and ligand motion are coupled for Xe migration through trHbN. Here, this was explicitly demonstrated for amino acids involved in the Xe1a↔Xe2 transition, and especially for the Phe^E15^ side chain. It is known from recent work [22, 24, 43–45] that Markov State Models (MSMs) containing protein degrees of freedom can be constructed. However, the purpose of the present work is not to construct a MSM, but to characterize the nature of Xe migration itself.

### Discussion

The present work suggests that for Xe migration through the network formed by the Xe-pockets (which are internal packing defects) in trHbN, the protein and ligand degrees of freedom are coupled. The analysis of the MD simulations demonstrated that even the diffusion of the weakly interacting Xe atom is coupled to the continuous structural rearrangement of trHbN as the ligand migrates through it. In fact, trHbN directs Xe migration to the extent that the probability of observing a specific transition (between states A and B) at a given time depends on where the ligand was before (state C). This was explicitly illustrated for Xe1a↔Xe2 events, where the amino acids composing the Xe1a↔Xe2 transition channel adopted different conformations depending on where Xe arrived from before the transition took place. The explicit MD simulations imply the presence of protein-induced memory effects through coupling between ligand and protein degrees of freedom, which greatly affect the migration of Xe. In other words, for a given transition, A→B, depending on where Xe comes to A from, trHbN rearranges differently.

The observation of memory effects raises the question whether, and if so, on what time scale ligand migration is a Markovian process. Based on time scale considerations [46, 47] a process is expected to be Markovian when one of its major sub-processes occurs on a considerably longer time scale with respect to other major sub-processes. For ligand migration, this would translate in transition times and intra-pocket relaxation occurring on different time scales. This should be possible when the free energy barriers separating the different pockets of the network are sufficiently high to ensure dwell times that are significantly longer than transition times, which is not the case in the present work.

While the assumption of Markovianity is commonly employed when describing chemical and biological processes, it has been known from several cases that it is not always valid [46, 48]. Characteristic examples of processes exhibiting non-Markovian behaviour are enzymatic reactions [49, 50] or the glass transition in polymers [51]. The true nature of the dynamics of such processes remains hidden unless appropriate reaction coordinates [46] or analysis techniques [48] are used for their investigation.

Ultimately, the preferable approach to probe the Markov-assumption is to address the problem in terms of probabilities. A ligand migration network is only Markovian if the Markov property holds for the transition probabilities between two pockets, A and B: *P*_(C→)A→B_ = *P*_(D→)A→B_. In this equation, C and D correspond to any two different pockets of the migration network from which the ligand can arrive to A. From a physical point of view, this equation states that the probability of observing A→B transitions is the same no matter from where the ligand arrived to A. That is, memory effects do not impact ligand diffusion through the protein.

However, as repeatedly shown in the present work, this is not found for Xe migration in trHbN. Rather, depending on the origin of the ligand (state C), the probability for the transition and the ensuing dynamics and conformations of the protein differ. The comparison of transition probabilities revealed that back-and forth events (B→A→B) are far more common than transitions C→A→B. Hence Xe migration in trHbN belongs to an expanding group of chemical and biological processes [46, 49–51] for which such an unbiased description reveals that the Markovianity assumption is not valid. The present study finds that Xe migration in trHbN is non-Markovian and occurs on a time scale of 10 to 20 ps. This time scale is identical to the one determined on a theoretical investigation of enantioselective reactions [52]. Moreover, the present work provides solid evidence that memory effects are operative in protein motion [53]. More precisely, for a specific event (transition), this translates to a different rearrangement of the amino acids involved in the transition region, depending on where Xe came from before the transition.

A question that arises is whether the present findings also apply to the migration of trHbN’s natural ligands, O_2_ and NO. Compared to Xe, both of these ligands have a different van der Waals shape, as well as the potential of interacting with the side chains of the amino acids that are involved in trHbN’s ligand migration network. Earlier computational investigations of O_2_, NO, CO and Xe migration in myoglobin [13] demonstrated that all four ligands localize in the same pockets and migrate along the same channels, but the free energy barriers separating these pockets are different for each ligand. For trHbN, Xe, NO and O_2_ migrate through the same pockets and transition channels, and the free energy barriers separating the pockets in NO and O_2_ migration [20, 21, 25–27, 29] (1-2 kcal/mol) are on the same order of magnitude as those for Xe migration determined in the present work. This suggests that the nature of Xe migration in trHbN is similar for NO and O_2_ migration in trHbN. Hence, it is expected that for the physiologically relevant NO and O_2_ ligands, migration is also non-Markovian but further investigation of this point is warranted to substantiate this.

Finally, it is also of interest to briefly touch on the question whether and how such effects could be observed experimentally. Potential experiments for the problem at hand require a spatial resolution of a few Å and a time resolution on the picosecond time scale. In addition, it will be advantageous to control the number of possible migration pathways through suitable point mutations as has already been successfully done for Myoglobin. [54] Time-resolved Laue diffraction allows characterization of structural changes on the sub-Å scale with a time resolution of 100 ps which is, however, probably too slow for the present purposes. [55] On the other hand, using different ligands (NO, CO) the dynamics may be slowed down due to increased barriers for ligand migration. X-ray free electron lasers (XFELs) offer new promising avenues to characterize the short-time dynamics of biological systems. It is expected that time-resolved serial femtosecond crystallography (SFX) and wide angle X-ray scattering (WAXS) at XFELs allow to investigate ultrafast protein structural dynamics on the femtosecond to picosecond time-scale. [56, 57] Alternatively, femtosecond X-ray solution scattering has been used to characterize the ultrafast increase of the radius of gyration of Mb on the 1 ps time scale. Such studies suggest that with ultrafast laser pulses the intrinsic motions in proteins can be characterized in the nonequilibrium (protein quake) regime. [58]

In summary, the migration of Xe in trHbN of Mycobacterium Tuberculosis was investigated using classical MD simulations. The analysis shows that Xe migration in trHbN occurs on the 10 to 20 ps time scale and is non-Markovian. In addition, the results demonstrate that memory effects are in operation during Xe motion through the internal protein pockets. For a specific transition, this translates into different rearrangements of the amino acids lining the transition region, depending on where Xe came from before. Whether or not this observation is physiologically relevant requires simulations with chemically active ligands such as NO or O_2_.

## Supporting information

S1 Text In this document, additional analysis of the simulations is reported. (PDF)
