# Supplementary material for: Migration of small ligands in globins: Xe diffusion in truncated hemoglobin N
Source: PLoS Comput Biol. 2017 Mar 30;13(3):e1005450. doi: 10.1371/journal.pcbi.1005450 (PMC5391117; doi:10.1371/journal.pcbi.1005450)
Supplement: S1 Text — (PDF) [file pcbi.1005450.s001.pdf]

Migration of Small Ligands in Globins: Xe Diffusion in Truncated Hemoglobin N: **Supporting Information**

Polydefkis Diamantis[1], Oliver T. Unke[1], Markus Meuwly[1¶]

1 Department of Chemistry, University of Basel, Basel, Switzerland

Xe Transitions from $x-$, $y-$ and $z-$ Coordinates

In Figure 1, similar to Figure 2 of the main text, Xe transitions are identified in a 5 ps trajectory, based on the evolution of its $x-$, $y-$ and $z-$ coordinates, and its distance from the heme's iron atom.

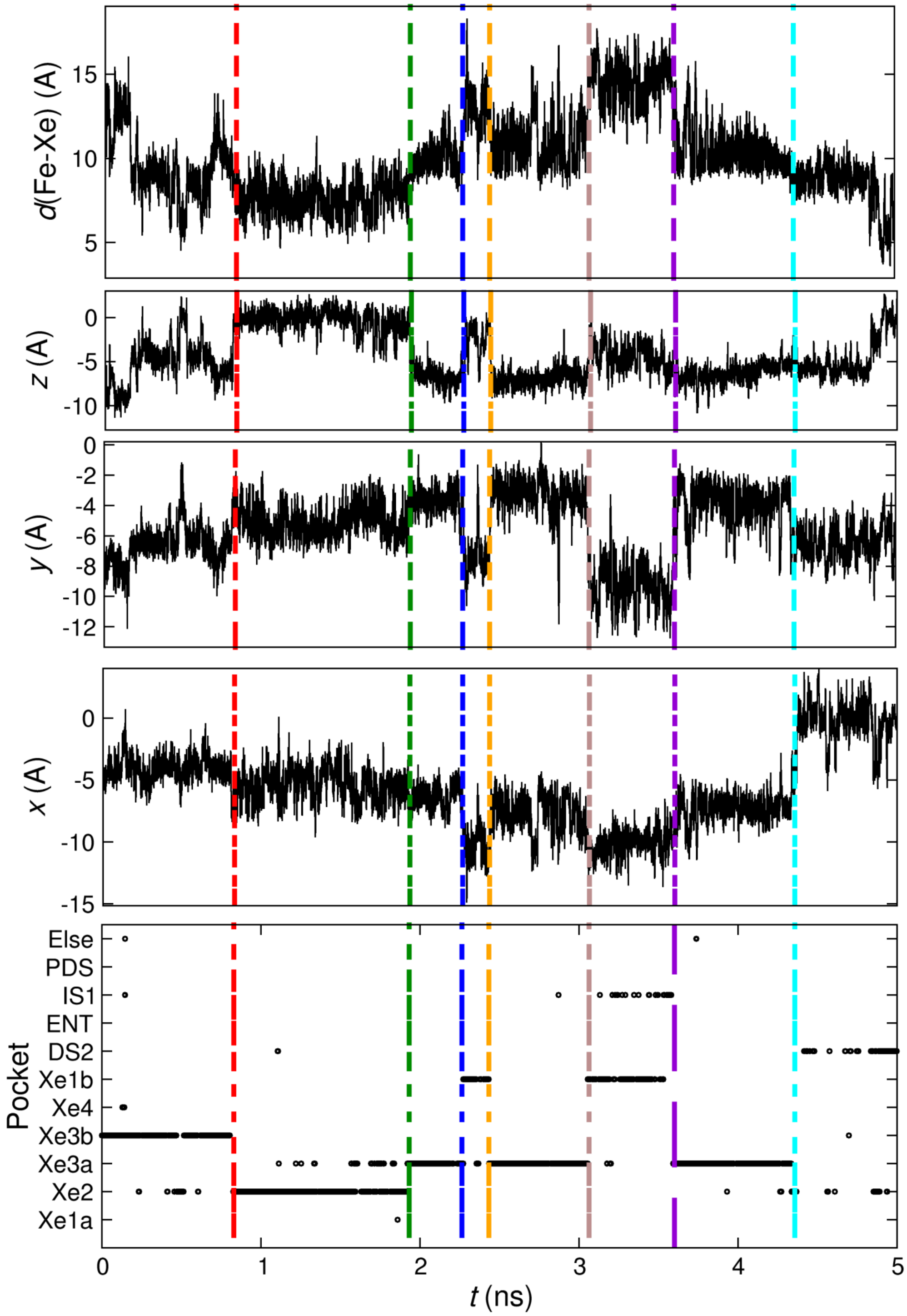

Fig 1. S1: Illustration of transitions in a 5 ns trajectory. Identification of Xe transitions by tracking the evolution of the Fe-Xe distance and $x-$, $y-$, and $z-$distances from origin (Fe atom), over 5 ns of explicit MD. The selected transitions are shown via vertical dashed lines.

Comparison of Dihedral Angle Distributions For Xe1a$\leftrightarrow$Xe2 Transitions

Similar to Figure 6 of the manuscript, the distributions of (i) the Cα-Cβ-Cγ-Cδ2 dihedral angle of Phe^{E15} and (ii) the distance of Xe from the Xe2 pocket upon Xe2$\rightarrow$Xe1a transitions, are shown in Figure 2.

Besides Phe^{E15}, the distributions of dihedral angles of Phe^{B9} (Cα-Cβ-Cγ-Cδ2), Leu^{G12} (Cα-Cβ-Cγ-Cδ1), Val^{B6} (C-Cα-Cβ-Cγ2) and Gln^{E11} (Cα-Cβ-Cγ-Cδ) upon Xe2$\leftrightarrow$Xe1a transitions were also determined and compared with the corresponding equilibrium distributions, shown in Figures 3 to 6.

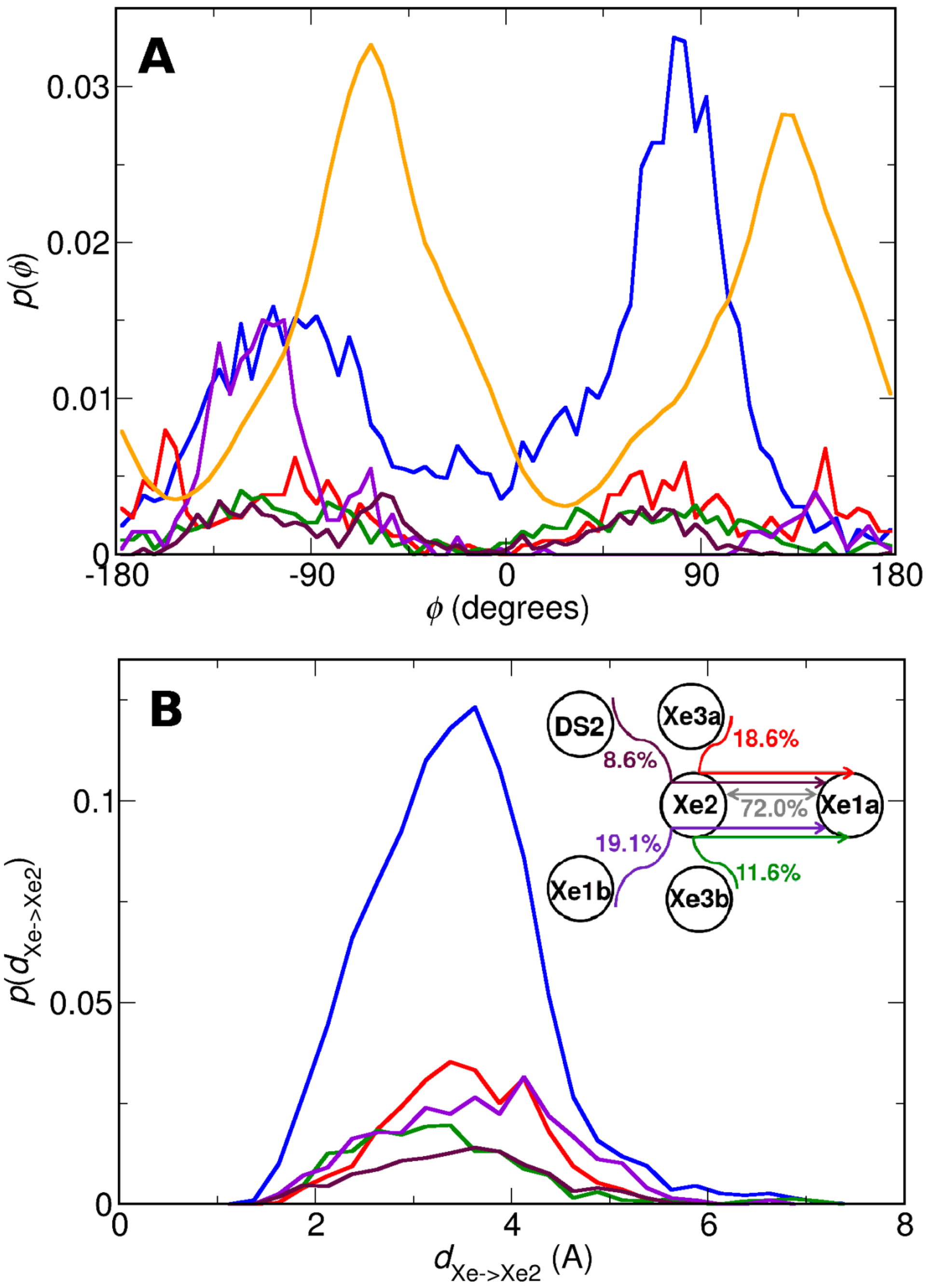

Fig 2. S2: Analysis of C→Xe2→Xe1a transitions. Distribution of the (A) Cα-Cβ-Cγ-Cδ2 dihedral angle of Phe^{E15} depending on state C in the transition C→Xe2→Xe1a. The equilibrium distribution (orange) is shown together with $P(\phi)$ for C=Xe1a (blue), C=Xe3a (red), C=Xe3b (green), C=Xe1b (violet) and C=DS2 (brown). (B) Xe distance from center of the Xe2 state for the Xe2→Xe1a transitions, from explicit MD simulations. Depending on where Xe entered the Xe2→Xe1a transition, the distributions differ.

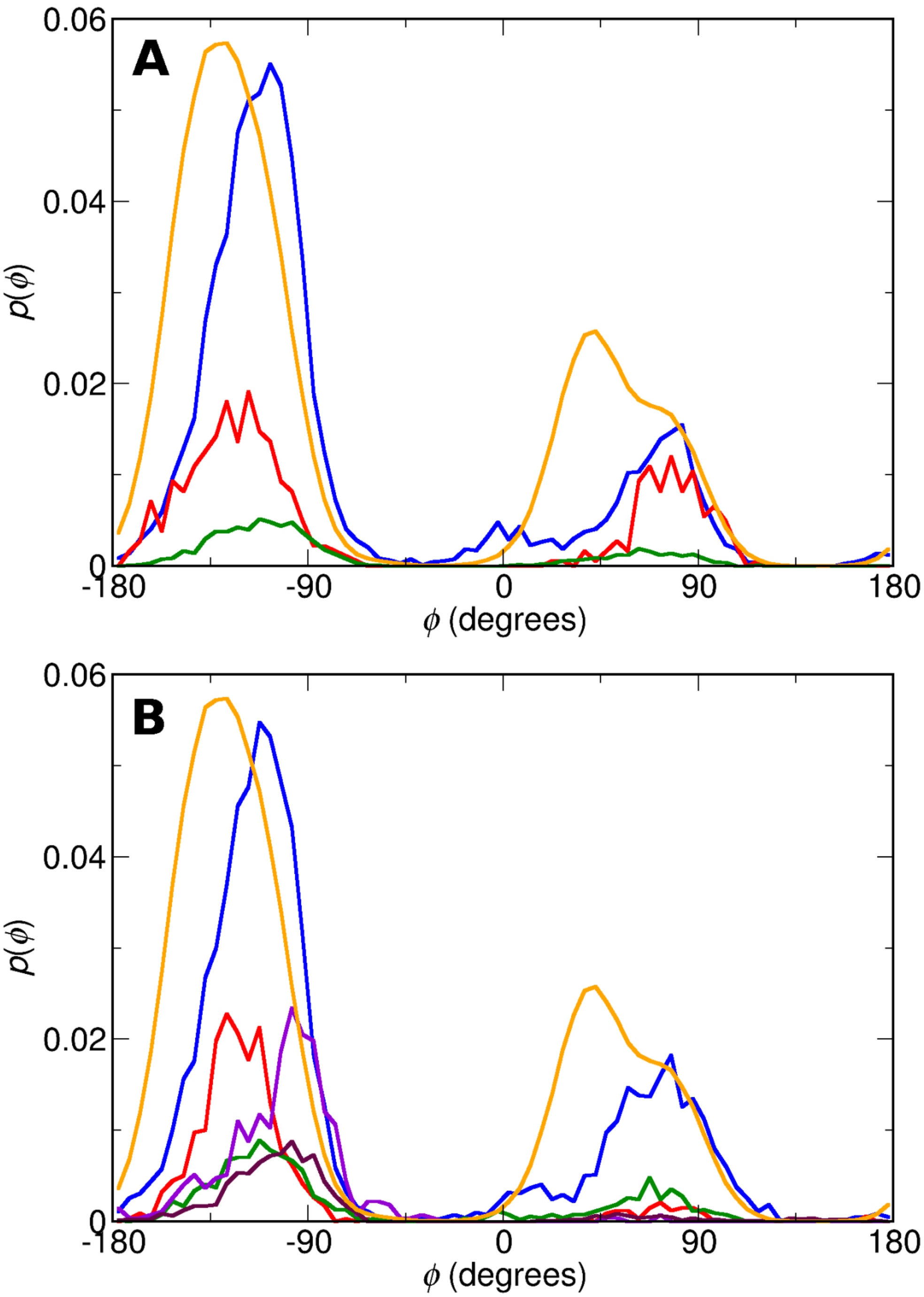

Fig 3. S3: Phe^{B9} dihedral angle distribution upon Xe1a→Xe2 and Xe2→Xe1a transitions. (A) Distribution of the Cα-Cβ-Cγ-Cδ2 dihedral angle of Phe^{B9} depending on state C in the transition C→Xe1a→Xe2. The equilibrium distribution (orange) is shown together with $P(\phi)$ for $P(\phi)$ for C=Xe2 (blue), C=Xe3a (red), C=Xe1b (green). (B) Same comparison, for C→Xe2→Xe1a transitions. The equilibrium distribution is compared with the distributions corresponding to C=Xe1a (blue), C=Xe3a (red), C=Xe3b (green), C=Xe1b (violet) and C=DS2 (brown).

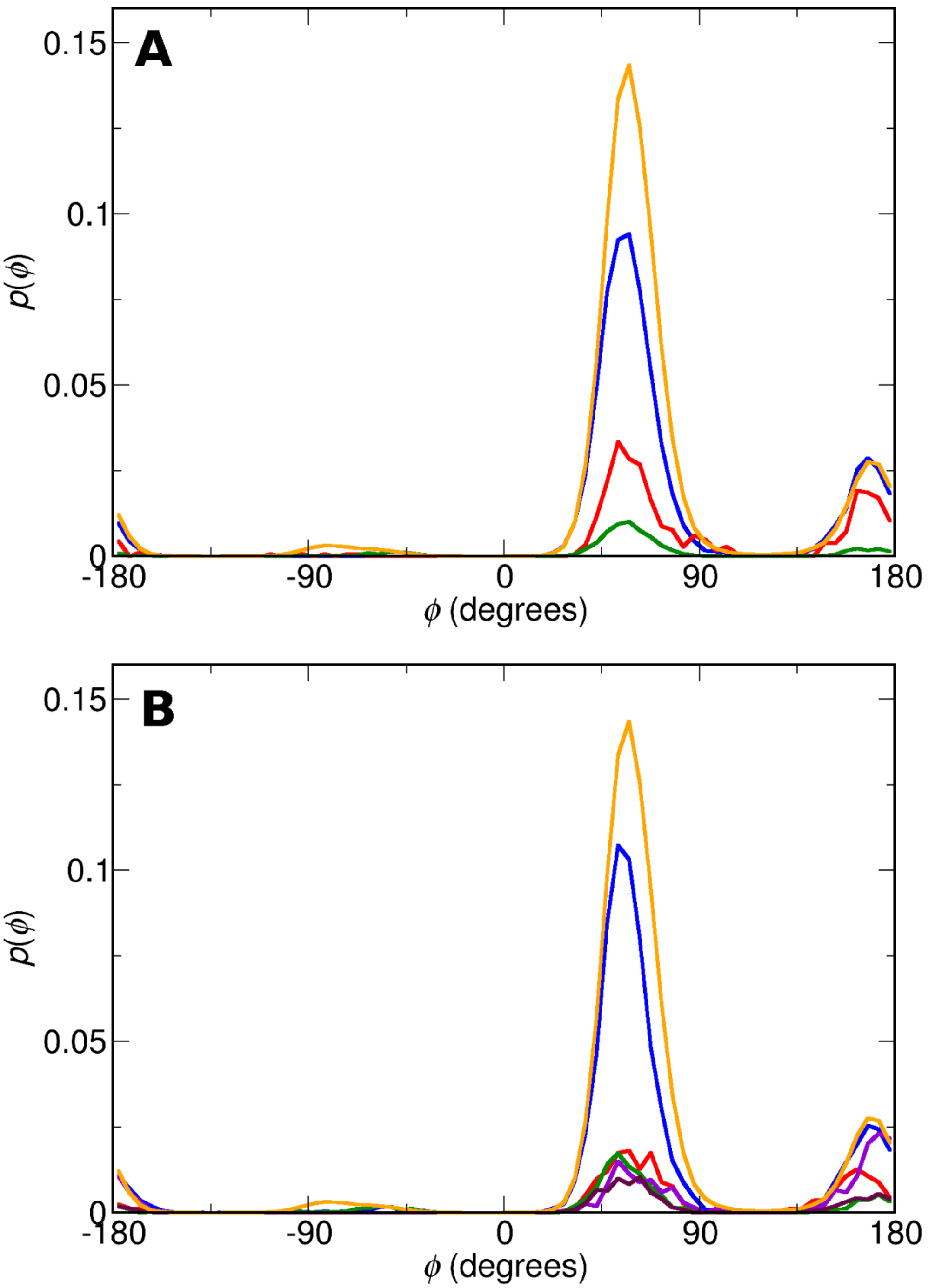

Fig 4. S4: Leu^{G12} dihedral angle distribution upon Xe1a→Xe2 and Xe2→Xe1a transitions. (A) Distribution of the Cα-Cβ-Cγ-Cδ1 dihedral angle of Leu^{G12} depending on state C in the transition C→Xe1a→Xe2. The equilibrium distribution (orange) is shown together with $P(\phi)$ for $P(\phi)$ for C=Xe2 (blue), C=Xe3a (red), C=Xe1b (green). (B) Same comparison, for C→Xe2→Xe1a transitions. The equilibrium distribution is compared with the distributions corresponding to C=Xe1a (blue), C=Xe3a (red), C=Xe3b (green), C=Xe1b (violet) and C=DS2 (brown).

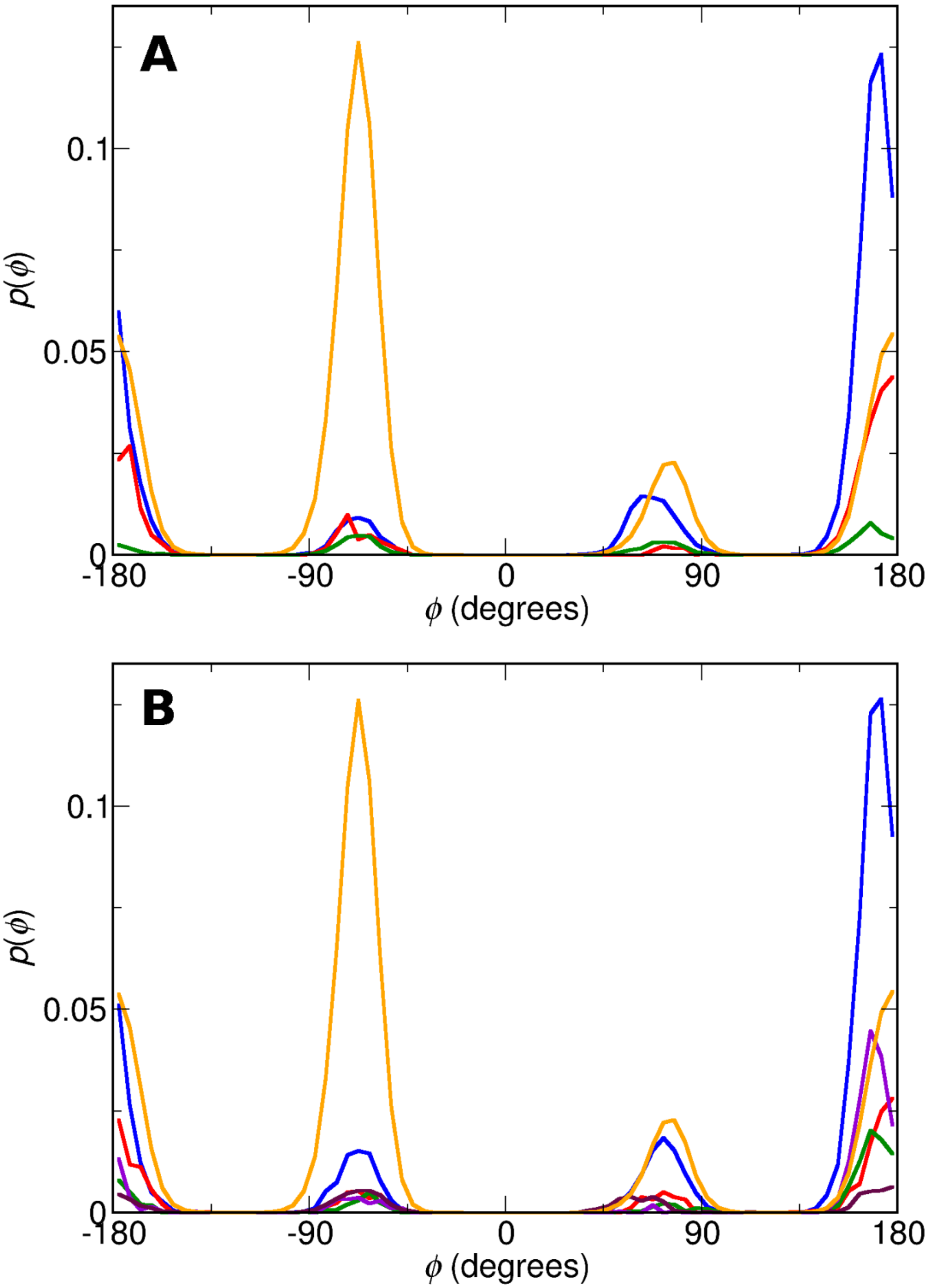

Fig 5. S5: Val^{B6} dihedral angle distribution upon Xe1a→Xe2 and Xe2→Xe1a transitions. (A) Distribution of the C-Cα-Cβ-Cγ2 dihedral angle of Val^{B6} depending on state C in the transition C→Xe1a→Xe2. The equilibrium distribution (orange) is shown together with $P(\phi)$ for $P(\phi)$ for C=Xe2 (blue), C=Xe3a (red), C=Xe1b (green). (B) Same comparison, for C→Xe2→Xe1a transitions. The equilibrium distribution is compared with the distributions corresponding to C=Xe1a (blue), C=Xe3a (red), C=Xe3b (green), C=Xe1b (violet) and C=DS2 (brown).

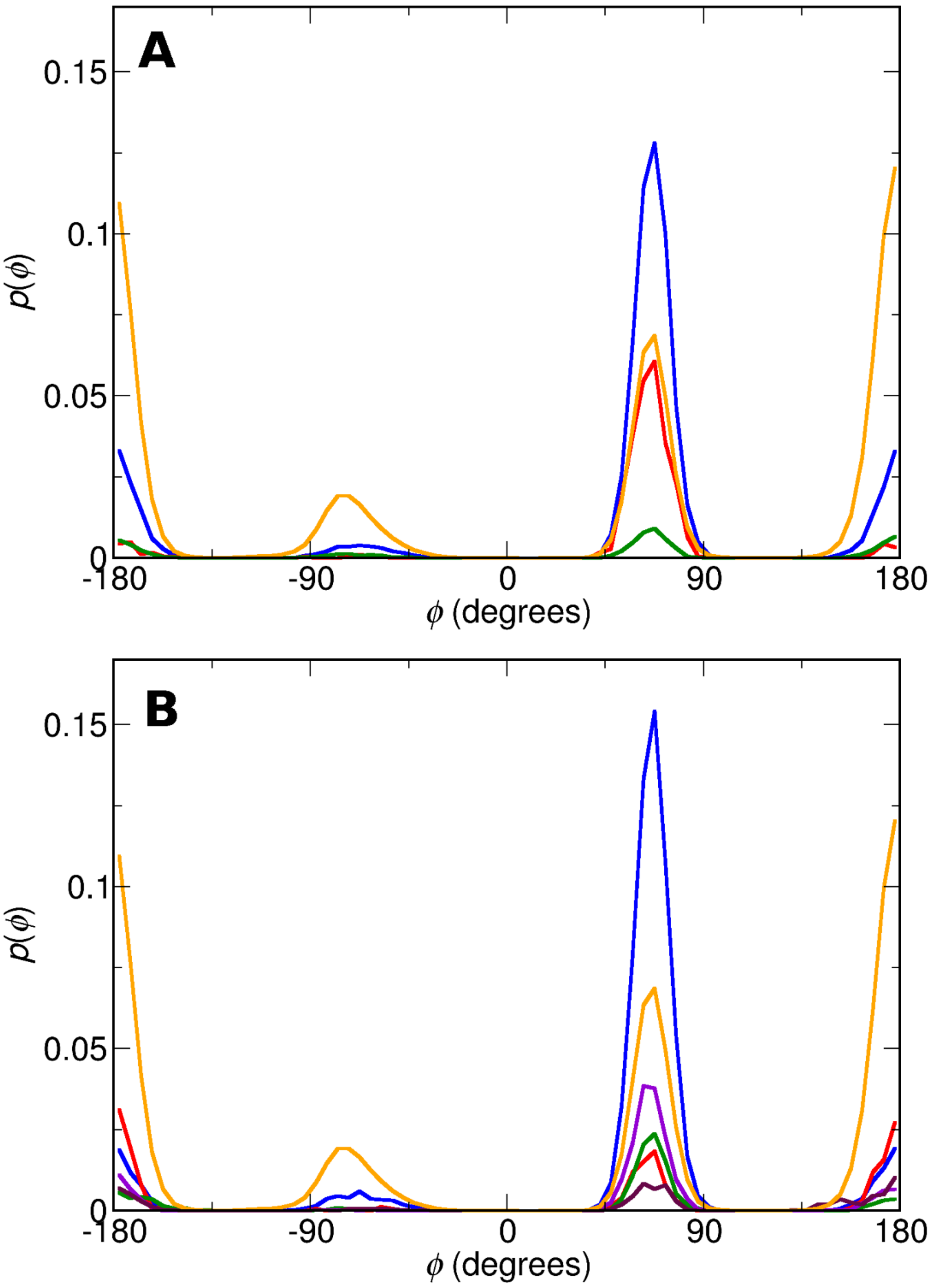

Fig 6. S6: Gln^{E11} dihedral angle distribution upon Xe1a→Xe2 and Xe2→Xe1a transitions. (A) Distribution of the Cα-Cβ-Cγ-Cδ dihedral angle of Gln^{E11} depending on state C in the transition C→Xe1a→Xe2. The equilibrium distribution (orange) is shown together with $P(\phi)$ for $P(\phi)$ for C=Xe2 (blue), C=Xe3a (red), C=Xe1b (green). (B) Same comparison, for C→Xe2→Xe1a transitions. The equilibrium distribution is compared with the distributions corresponding to C=Xe1a (blue), C=Xe3a (red), C=Xe3b (green), C=Xe1b (violet) and C=DS2 (brown).

Xe and Amino Acid Atom Projections upon Xe2→Xe1a Transitions

Xe and amino acid atom positions corresponding to Xe2→Xe1a transitions were also projected on the same plane used for Xe1a→Xe2 transitions. The resulting graphs are of the same type as those of Figure 7C of the manuscript, and are depicted in Figure 7.

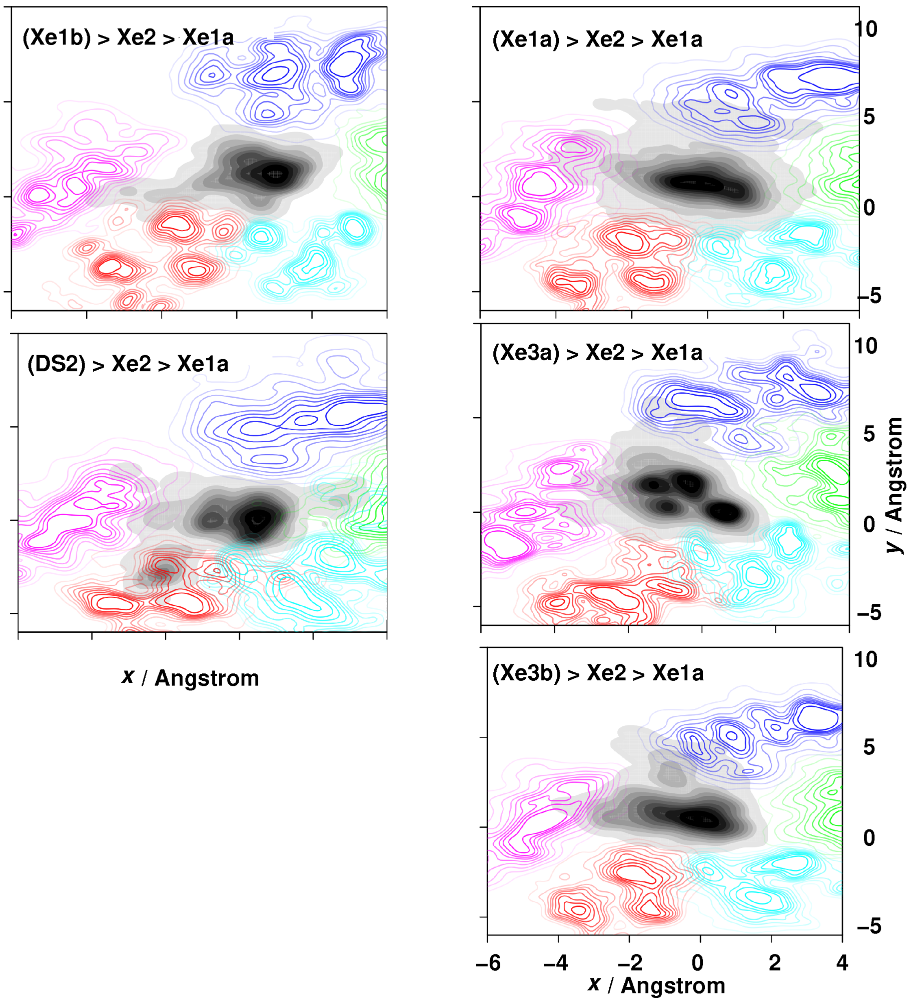

Fig 7. S7: Difference in projection of Xe and amino acid atom positions upon C→Xe2→Xe1a transitions. Projection of the position of Xe (greyscale) and Val^{B6} (cyan), Phe^{B9} (green), Gln^{E11} (red), Phe^{E15} (magenta) and Leu^{G12} (blue) atoms onto the plane containing the transition points for different C→Xe2→Xe1a transitions. C corresponds to Xe1a, Xe3a, Xe3b, Xe1b or DS2. The projections are presented via density isocontours. The integrated density corresponds to 100%. Moving from inner to outer contours, each of them contains an additional 10% of the total number of points. For the definition of the transition plane see Figure 7 in the main text.
